# Does Helping Others Always Benefit Health? Longitudinal Evidence on the Relationship between Helping Behavior and Depression: The Mediating Role of Life Satisfaction and the Moderating Effect of IADL

**DOI:** 10.1155/2024/2304723

**Published:** 2024-07-25

**Authors:** Yan Cheng, Yue Wei, Shao-Liang Tang

**Affiliations:** ^1^Nanjing Hospital of Chinese Medicine Affiliated to Nanjing University of Chinese Medicine, Nanjing 210000, China; ^2^Nanjing University of Chinese Medicine, Nanjing 210023, China

## Abstract

**Background:**

This study aims to explore whether helping behavior is always beneficial for alleviating depression or if there is a “moderation is the key” effect.

**Materials and Methods:**

This study focused on a sample of 7,436 participants from the China Health and Retirement Longitudinal Study (CHARLS). The 10-item Center for Epidemiologic Studies Depression Scale (CESD-10) was used to identify the presence of depression. Linear mixed model and Quasi-Bayesian estimation methods were used to explore the mediating role of life satisfaction in the relationship between helping behavior and depression, as well as the moderating effects of the instrumental activity of daily living (IADL). Additionally, we employed the Johnson–Neyman technique to visualize the moderating effect of IADL.

**Results:**

Helping behavior shows a negative correlation with depression (*B* = −0.170, *p* = 0.020), where life satisfaction fully mediates this relationship (effect = −0.055, 95% confidence interval = −0.088 to −0.022). Moreover, the association between helping behavior and life satisfaction is moderated by IADL (*B* = −0.047, *p* < 0.001). Specifically, when IADL is below 0.56, helping behavior positively impacts life satisfaction. In contrast, when IADL exceeds 1.99, helping behavior has a detrimental effect on life satisfaction.

**Conclusions:**

This study highlights the significant positive impact of helping behavior on depression alleviation, which is achieved by increasing life satisfaction. Notably, although helping behavior has positive effects on individuals, not everyone can benefit directly from it. Only those without functional limitations are more likely to experience the benefits of such behavior. Therefore, when policymakers and researchers develop strategies to encourage individuals in helping behavior to combat depression, they should consider two key approaches. First, life satisfaction should be used as an important indicator in the treatment of depression, allowing for timely adjustments to ensure the effectiveness and individualization of treatment plans. Second, the principle of “moderation is the key” should be prioritized, ensuring that helping behavior can maximize its benefits and help individuals emerge from the shadows of depression.

## 1. Introduction

With the exacerbation of aging, depression has become a significant public health concern threatening the life and health of the Chinese population. According to statistics, the lifetime prevalence of depressive disorders among Chinese adults is 6.8%, yet only 0.5% of patients received adequate treatment [1]. The persistently high prevalence brings an unavoidable economic burden. In China, absenteeism, medical expenses, and funeral costs related to depression are estimated to cause a loss of $7.8 billion annually [2]. Therefore, depression is a major health issue in China that cannot be ignored. It has already caused and is further exacerbating the social and economic burden on the nation and requires further attention.

Previous research has found a close association between depression and various factors such as lifestyle habits [3, 4, 5], physical health status [6, 7, 8, 9], and indoor/outdoor air quality [10, 11]. In recent years, an increasing number of studies using nationally representative large-scale public databases have found significant advantages of helping behavior in maintaining health. This behavior not only reduces blood pressure [12], benefits in cognitive functioning [13], and delays the progression of frailty [14] but also reduces the risk of Alzheimer's disease [15]. It can even counteract the adverse effects on the mental health of parents caused by the loss of their children [16], reduce the likelihood of cognitive decline in individuals with a high genetic risk of Alzheimer's disease [17], and decrease prescription medication use for pain and depression [18]. Particularly in alleviating depression, this behavior demonstrates unique therapeutic effects [16, 19, 20, 21, 22]. However, there are still relatively few studies that use nationally representative longitudinal data to explore the impact of helping behavior on the health of individuals in the Chinese population.

Moreover, previous studies have identified life satisfaction as a crucial mediator in the relationship between depression and a wide range of influencing factors, including chronic diseases [23, 24], economic [25] and energy poverty [26], childhood socioeconomic status [27], childhood maltreatment [28], social capital [29], and internet use [30]. These studies define life satisfaction as an individual's overall evaluation of their current life experiences and attitudes. They argue that individuals with higher life satisfaction tend to live independently, actively participate in social activities, and possess a stronger sense of self-worth, which serves as a significant protective factor against perceived stress. Additionally, Liu et al. [31] have summarized previous research and pointed out that subjective life satisfaction is closely linked to individual development. Individuals with a high level of life satisfaction tend to exhibit greater self-efficacy, lower stress levels, and more harmonious interpersonal relationships, all contributing to their overall health. However, despite this significant insight, there is currently a lack of research exploring the influence of helping behavior on depression among the Chinese population, taking life satisfaction as a mediating factor.

Since helping behavior, like physical activity and internet use, is a common and self-manageable behavior in daily life. Additionally, helping behavior is an important part of social participation [32], which has been widely proven to be a healthy behavior. Therefore, we can view helping behavior as similar to physical activity and internet use, belonging to the category of health behaviors with health-promoting effects. In the field of health behavior research, multiple studies have shown the existence of the “moderation is the key” effect. That is, moderate behavior is beneficial to health, whereas excessive behavior may lead to adverse health effects. For instance, moderate sleep, physical activities, and internet use are beneficial to health, but insufficient or excessive sleep [33], excessive physical activities [34], and internet use [30, 35] can weaken these benefits or even have adverse health consequences. In previous research, a small body of literature has found that moderate frequencies of helping behavior are beneficial to health, while both less and more frequent helping behavior are not significant [14, 20]. This suggests that helping behavior may also follow the principle of “moderation is the key,” which warrants further investigation.

In 2015, the World Health Organization proposed the concept of healthy aging, which is defined as the process of developing and maintaining the functional ability that enables well-being in older age. Intrinsic capacity refers to the combination of all physical and mental abilities that individuals can draw upon at any time to accomplish what they consider important [36]. In many previous studies [37, 38, 39, 40], instrumental activity of daily living (IADL) has been regarded as an important component in assessing an individual's intrinsic capacity. At the same time, the content assessed by IADL is closely related to helping behavior. For example, providing assistance to relatives or friends requires cooking and housekeeping skills; participating in volunteer or charity activities requires financial management skills; and providing unpaid care to patients or disabled individuals in different residential areas requires the ability to ensure patients take medication on time. Therefore, we believe that from the perspective of functional performance, IADL can be used to explore whether helping behavior follows the principle of “moderation is the key.”

**Figure 1:**
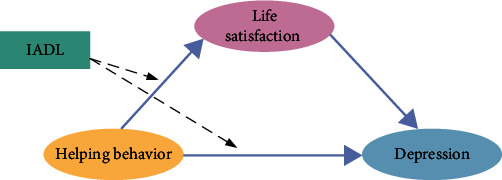
The conceptual moderating mediation model. *Note*. IADL, instrumental activity of daily living.

This study aims to explore the potential role of helping behavior in alleviating depression among the Chinese population and to investigate whether life satisfaction mediates this relationship. Furthermore, we aim to explore whether helping behavior is influenced by an individual's IADL. In other words, whether the impact of this behavior on life satisfaction and depression varies according to differences in an individual's IADL level. We provide three hypotheses for this research in light of those above, as seen in Figure 1.

Hypothesis 1: In the Chinese population, helping behavior is negatively associated with depression. Studying this hypothesis is beneficial for us to initially explore the total effect between helping behavior and depression, providing a basis for investigating the potential mediating role of life satisfaction and the moderating effect of IADL in subsequent research, thereby further revealing its underlying mechanisms.

Hypothesis 2: In the Chinese population, life satisfaction is a key mediating factor between helping behavior and levels of depression. Studying this hypothesis helps us uncover the mechanisms by which helping behavior influences mental health, providing a new perspective for enhancing the quality of life among the Chinese population.

Hypothesis 3: Helping behavior follows the principle of “moderation is the key.” That is, when there are no functional limitations, helping behavior is beneficial to individuals' health. However, when functional limitations exist, helping behavior no longer benefits individuals' health and may even have adverse effects. Studying this hypothesis helps us understand the mechanisms by which functional limitations affect the mental health of the Chinese population, providing a reference for developing personalized intervention measures.

## 2. Materials and Methods

### 2.1. Data Sources and Participation

China Health and Retirement Longitudinal Study (CHARLS) aims to gather high-quality microdata that represents Chinese individuals and households aged 45 and above. These data are crucial for analyzing the challenges posed by an aging population in China and promoting interdisciplinary research on aging. The survey was conducted in five waves: 2011 (Wave 1), 2013 (Wave 2), 2015 (Wave 3), 2018 (Wave 4), and 2020 (Wave 5), covering 150 counties and 450 communities across 28 provinces, autonomous regions, and municipalities [41]. The study also includes PM2.5 data obtained from the Atmospheric Composition Analysis Group (ACAG) at Dalhousie University, which used satellite and ground monitoring stations to provide detailed information on PM2.5 levels [42]. The research will utilize the combined data from CHARLS W1-W5, the Harmonized CHARLS (Version D), and the ACAG for further empirical analysis.

**Figure 2:**
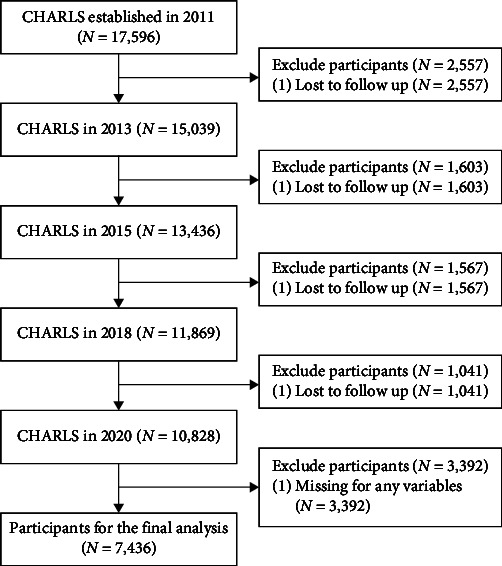
Flowchart of participant selection.

A total of 17,596 participants were included in Wave 1 of this study. During the sample selection process, In the follow-up surveys of Waves 2–5, 2,557, 1,603, 1,567, and 1,041 participants were lost to follow-up or deceased, respectively. Additionally, we excluded 3,392 participants with any missing values. Finally, a total of 7,436 participants were included in the analysis (Figure 2).

### 2.2. Measures

#### 2.2.1. Outcome Variable: Depression

In CHARLS, depression was measured using the Chinese version of the 10-item Center for Epidemiologic Studies Depression Scale (CESD-10). Participants were asked to rate how often they experienced depressive symptoms in the past week, from “rarely” to “some days” (1–2 days), “occasionally” (3–4 days), or “most of the time” (5–7 days). The CESD-10 consists of 10 questions, with each question scored from 0 to 3. The total scale score ranges from 0 to 30, with higher scores generally indicating greater severity of depression.

#### 2.2.2. Exposure Variable: Helping Behavior

Based on previous research [32, 43, 44, 45, 46] that utilized nationally representative large-scale public databases to evaluate whether helping behavior is beneficial to health, in CHARLS, we defined the following behaviors as helping behavior: (1) provided help to family, friends, or neighbors who do not live with participants; (2) done voluntary or charity work; and (3) cared for a sick or disabled adult who does not live with participants. If participants engaged in any of the above behaviors, they were considered to have engaged in helping behavior.

#### 2.2.3. Mediator Variable: Life Satisfaction

Participants were asked to rate their life satisfaction from “not at all satisfied” to “completely satisfied.” The total range of life satisfaction scores in this study was 0–5, with higher scores indicating greater satisfaction.

#### 2.2.4. Moderator Variable: IADL

IADL includes doing housework, cooking, shopping, taking medicine, and managing finances. All items have four options: “no difficulty,” “difficulty but can complete,” “difficulty and need help,” and “unable to complete.” We collapsed the above options into two categories: “no difficulty” and “difficulty.” Finally, the total range of IADL scores in this study was 0–5, which indicated the total number of physical limitations.

#### 2.2.5. Covariates

We included demographic characteristics as potential confounding covariates, along with factors that have been clearly associated with depression in previous research targeting the Chinese population. Demographic characteristics include age, urban–rural disparities, gender, education, and marital status. Potential influencing factors include chronic disease [7], falls [9], pain [8], self-rated health [47], indoor air pollution [11], outdoor air pollution [10], drinking, smoking [48], sleep duration [4], and internet use [30].

### 2.3. Statistical Analyses

Continuous variables are described using the mean and standard deviation (SD), while categorical variables are described using frequency and percentage.

We constructed a linear mixed model (LMM) to investigate the relationship between the critical variables (helping behavior, life satisfaction, IADL, and depression). We referred to previous studies and treated ID as a random intercept, thus reflecting the inherent differences between individuals at baseline levels. Meanwhile, the survey time points were treated as random slopes to capture the trends of individuals' responses over time [49, 50]. Finally, we employed the residual maximum likelihood method to estimate the parameters of the model [51].

Mediation analyses were performed using the Quasi-Bayesian estimation of 95% confidence intervals (CIs) and 2,000 Monte Carlo iterations, with a 95% CI that did not contain 0, indicating a significant mediating effect [52].

The design of the specific model is as follows:

(1) $$\begin{array}{c} \text{Depressio}n_{ij}=\beta_{0}+\beta_{1}\cdot \text{Helpin}g_{ij}+\beta_{2}\cdot {\text{wave}}_{ij}+\sum_{k}\gamma_{k}\cdot \text{ControlVa}r_{kij}+u_{0i}+u_{1i}\cdot {\text{wave}}_{ij}+\varepsilon_{ij}, \end{array}$$

(2) $$\begin{array}{c} \text{LifeSa}t_{ij}=\beta_{0}+\beta_{1}\cdot \text{Helpin}g_{ij}+\beta_{2}\cdot {\text{wave}}_{ij}+\sum_{k}\gamma_{k}\cdot \text{ControlVa}r_{kij}+u_{0i}+u_{1i}\cdot {\text{wave}}_{ij}+\varepsilon_{ij}, \end{array}$$

(3) $$\begin{array}{c} \text{Depressio}n_{ij}=\beta_{0}+\beta_{1}\cdot \text{Helpin}g_{ij}+\beta_{2}\cdot \text{LifeSa}t_{ij}+\beta_{3}\cdot {\text{wave}}_{ij}+\sum_{k}\gamma_{k}\cdot \text{ControlVa}r_{kij}+u_{0i}+u_{1i}\cdot {\text{wave}}_{ij}+\varepsilon_{ij}, \end{array}$$

(4) $$\begin{array}{c} \text{LifeSa}t_{ij}=\beta_{0}+\beta_{1}\cdot \text{Helpin}g_{ij}+\beta_{2}\cdot {\text{IADL}}_{ij}+\beta_{3}\cdot \left(\text{Helpin}g_{ij}\cdot {\text{IADL}}_{ij}\right)+\beta_{4}\cdot {\text{wave}}_{ij}+\sum_{k}\gamma_{k}\cdot \text{ControlVa}r_{kij}+u_{0i}+u_{1i}\cdot {\text{wave}}_{ij}+\varepsilon_{ij}, \end{array}$$

(5) $$\begin{array}{c} \text{Depressio}n_{ij}=\beta_{0}+\beta_{1}\cdot \text{LifeSa}t_{ij}+\beta_{2}\cdot \text{Helpin}g_{ij}+\beta_{3}\cdot {\text{IADL}}_{ij}+\beta_{4}\cdot \left(\text{Helpin}g_{ij}\cdot {\text{IADL}}_{ij}\right)+\beta_{5}\cdot {\text{wave}}_{ij}+\sum_{k}\gamma_{k}\cdot \text{ControlVa}r_{kij}+u_{0i}+u_{1i}\cdot {\text{wave}}_{ij}+\varepsilon_{ij}. \end{array}$$

Equation (1) examines the total effect of helping behavior on depression. Equation (2) examines the effect of helping behavior on life satisfaction. Equation (3) examines the effect of helping behavior and life satisfaction on depression (with life satisfaction as a mediator). Equation (4) examines the moderating effect of IADL on the relationship between helping behavior and life satisfaction. Equation (5) examines the moderating effect of IADL on the relationship between helping behavior and depression. In the above models, Depression represents the level of depression, Helping represents helping behavior, LifeSat represents life satisfaction, IADL represents instrumental activities of daily living, wave represents time points (2011, 2013, 2015, 2018, and 2020), ControlVar represents control variables, *β* represents fixed effect parameters, *γ* represents coefficients of control variables, *u*_0_ represents random intercepts, *u*_1_ represents random slopes, and *ε* represents error terms. Finally, we tested the moderated mediation to reveal the indirect effects of helping behavior through life satisfaction on depression at different levels of IADL (1 SD below the mean, the mean, and 1 SD above the mean) by using the Quasi-Bayesian estimation method. We also used the Johnson–Neyman technique to visualize the moderating effect of IADL on the relationship between helping behavior and life satisfaction or depression.

This analysis was performed on the complete cases data set, and a sensitivity analysis was performed using multiple imputations (MI) due to missing data [53]. Additionally, in the latest nationally representative epidemiological study of mental disorders in the Chinese population, it was found that the prevalence of major depressive disorder is significantly higher among individuals aged 50 and above, such as those aged 50–64 (OR 2.27 (95% CI 1.64–3.14)) and those aged 65 and above (OR 2.13 (95% CI 1.39–3.26)) [1]. Therefore, we conducted another sensitivity analysis specifically targeting individuals aged 50 and above. Furthermore, we conducted variance inflation factor (VIF) tests to examine the issue of multicollinearity in the models. The VIF test results showed that the VIF values for all variables in the models were much lower than the critical value of 10, indicating the absence of severe multicollinearity issues (see Table S1 for the VIF test).

Data processing and analysis were conducted using R software (Version 4.3.3, R Foundation for Statistical Computing, Vienna, Austria). The lme4 package was used to build the LMM [54], and the mediation package [55] and interactions package [56] were used to conduct the mediating and moderating effect analyses. The mice package [57] was used for MI to fill in the missing data, and the car package [58] was used to compute the VIF. A significance level of *p*  < 0.05 was used to indicate the statistical significance of differences.

## 3. Results

### 3.1. Descriptive Statistics

Table S2 summarizes the baseline characteristics of the participants. The study included participants with an average age of 56.40 (SD = 8.15). Among them, males accounted for 46.3% of the total sample, while the urban population represented 33.8% of the participants. Most participants were married (91.6%) and reported primary education (88.6%). Their mean score of depression was 8.12 (SD = 6.16), the average IADL score was 0.29 (SD = 0.79), and the average life satisfaction score was 3.05 (SD = 0.69). Most participants (90.7%) did not have helping behavior.

### 3.2. Mediating Effect of Life Satisfaction between Helping Behavior and Depression

As LMM showed, helping behavior was negatively associated with depression (*B* = −0.170, *p* = 0.020) (see Model 1 in Table 1). Hypothesis 1 has been validated. Helping behavior was positively associated with life satisfaction (*B* = 0.034, *p* < 0.001) (see Model 2 in Table 1). When simultaneously putting helping behavior and life satisfaction into the LMM, only life satisfaction (*B* = −1.614, *p* < 0.001) was significantly negatively associated with depression (see Model 3 in Table 1).

The results of the Quasi-Bayesian estimation method showed that the total effect (effect = −0.173, 95% CI = −0.314 to −0.027) and the indirect effect (effect = −0.055, 95% CI = −0.088 to −0.022) were significant, while the direct effect was not significant. This indicates that life satisfaction completely mediated the relationship between helping behavior and depression (see Table 2). Hypothesis 2 has been validated.

### 3.3. Moderating Effect of IADL

Since the previous section demonstrated the complete mediation effect of life satisfaction between helping behavior and depression, we solely examined the moderating effect of IADL on the relationship between helping behavior and life satisfaction. The results indicated a significant interaction effect (*B* = −0.047, *p*  < 0.001), suggesting that IADL moderates the relationship between helping behavior and life satisfaction (see Model 4 in Table 1).

**Figure 3:**
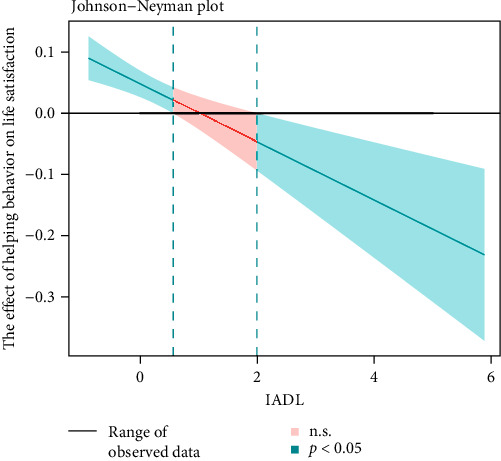
The Johnson−Neyman plot. *Note*. IADL, instrumental activity of daily living.

The results of the conditional indirect effect of helping behavior on depression via life satisfaction at different levels of IADL are presented in Table 2. The indirect effects were significant in low (effect = −0.114, 95% CI = −0.159 to −0.070) and mean (effect = −0.049, 95% CI = −0.080 to −0.020) levels of IADL, whereas the indirect effect was not significant in the high level of IADL. The Johnson–Neyman technique showed that when IADL is below 0.56 (indicating no restrictions), helping behavior has a significantly positive effect on life satisfaction; when IADL ranges between 0.56 and 1.99 (indicating one limitation), the effect of helping behavior on life satisfaction is no longer significant; when IADL is above 1.99 (indicating two or more limitations), helping behavior instead has a significantly negative effect on life satisfaction (Figure 3). Hypothesis 3 has been validated.

We conducted two sets of sensitivity analyses, one focusing on MI data and the other on individuals aged 50 and above. The sensitivity analyses results were consistent with the main findings. In the sensitivity analysis for MI data, life satisfaction partially mediated the relationship between helping behavior and depression. Therefore, we also examined the moderating effects of IADL on the relationship between helping behavior and life satisfaction, as well as between helping behavior and depression. The results showed that both of these moderating effects were significant (see Figure S1 for the final moderated mediation model). Johnson–Neyman plots clearly illustrate the effects of helping behavior on life satisfaction and depression at different levels of IADL: when IADL is less than 0.87, helping behavior contributes to increased life satisfaction; when IADL is less than 0.30, helping behavior helps alleviate depression. However, when IADL is between 0.87 and 3.06, helping behavior no longer benefits life satisfaction; when IADL is between 0.30 and 3.93, helping behavior no longer helps alleviate depression. It is worth noting that when IADL exceeds 3.06, helping behavior negatively affects life satisfaction. Similarly, when IADL exceeds 3.93, helping behavior instead worsens depression (see Figures S2 and S3 for Johnson–Neyman plots). In the sensitivity analysis for individuals aged 50 and above, life satisfaction fully mediated the relationship between helping behavior and depression, and IADL significantly moderated the relationship between helping behavior and life satisfaction (see Figure S4 for the final moderated mediation model). Johnson–Neyman plots clearly illustrate the effects of helping behavior on life satisfaction and depression at different levels of IADL: when IADL is less than 0.46, helping behavior contributes to increased life satisfaction; when IADL exceeds 0.46, helping behavior no longer contributes to increased life satisfaction (see Figure S5 for Johnson–Neyman plots).

## 4. Discussion

This study, for the first time, utilized a nationally representative large-scale public database to investigate the longitudinal association between helping behavior and depression in the Chinese population and further examined the mediating and moderating effects of life satisfaction and IADL in this association. Our results showed that helping behavior was negatively associated with depression, and this association was completely mediated by life satisfaction. Additionally, we found that helping behavior exhibits a “moderation is the key” effect. Specifically, when there were no limitations in IADL, helping behavior could enhance life satisfaction. However, when there was one limitation in IADL, helping behavior no longer promoted life satisfaction. Conversely, when there were two or more limitations in IADL, helping behavior inhibited life satisfaction.

Our study found a positive association between helping behavior and life satisfaction, consistent with numerous previous studies [59, 60, 61]. This may be because helping behavior can have multifaceted effects: First, helping behavior is beneficial for the physical health of individuals, such as delaying the progression of frailty [46], reducing cognitive risks [62], and relieving pain [63], thereby enhancing life satisfaction. Second, engaging in helping behavior not only can evoke positive aging-related emotions [32] but can also transform individuals from being “needy” to “needed,” allowing them to achieve a sense of self-worth and meaning in life [43], thus enhancing life satisfaction. Finally, this behavior is a part of social engagement [32], making individuals more popular and easily gaining support and care in society. It helps to establish intimate relationships and social support networks between individuals and others, increasing their social identity and sense of belonging, thereby enhancing their happiness.

Our study found a negative association between life satisfaction and depression, consistent with numerous previous studies [64, 65, 66, 67, 68, 69]. This may be due to several reasons: First, according to the emotional contagion theory, the happiness of individuals can be spread to family, friends, or community members through social interaction, creating a positive emotional atmosphere [70], which helps alleviate depression in individuals. Second, life satisfaction can help in coping more effectively with various difficulties and setbacks by maintaining inner peace and enhancing resilience. This positive mindset and resilience can reduce the generation of negative emotions and lower the risk of depression [71].

Our study further found that life satisfaction played a complete mediating role in the relationship between helping behavior and depression. Although there is currently a lack of research examining the relationship between helping behavior and depression with life satisfaction as a mediating variable, numerous previous studies have indicated that life satisfaction is an important mediating factor influencing depression [23, 24, 25, 26, 27, 28, 29, 30]. This suggests that increasing life satisfaction is a key factor in alleviating depression. Therefore, by enhancing life satisfaction, helping behavior can alleviate depressive symptoms, providing a new intervention approach for the mental health of individuals.

Our study also reveals a significant effect of helping behavior characterized by “moderation is the key.” This finding aligns with the “Goldilocks Principle,” which has wide applications in the medical field, covering various aspects such as disease screening and diagnosis [72], pharmacotherapy [73], nutritional therapy [74], surgical treatment [75], and exercise rehabilitation [76]. The Goldilocks Principle emphasizes the importance of “not too much, not too little, just right,” aiming to find the optimal balance point under specific conditions, ensuring patients receive the most appropriate treatment while avoiding potential unnecessary risks. This principle provides a new perspective for understanding the impact of helping behavior on life satisfaction and offers a theoretical basis for devising more personalized intervention strategies.

In our study, this balance point is whether there is a functional limitation. Specifically, when individuals have no functional limitations, their helping behavior can lead to an increase in life satisfaction, thereby helping to alleviate depression. This may be because they have sufficient intrinsic abilities to engage in social activities, thereby enhancing life satisfaction. However, when individuals have functional limitations, they may no longer be able to care for others or participate in volunteer or charity activities as before. In this case, if help is provided beyond their capabilities, what was once voluntary behavior will turn into a forced one, and their proactive attitude will become passive, imposing greater burdens on the individuals, leading to feelings of frustration and stress, thereby reducing their life satisfaction.

Furthermore, Liu et al. [31] clearly point out in their study that although traditional Confucian culture has left a profound imprint on Chinese social culture when conducting in-depth research, we must closely integrate the background of the current national conditions and cultural integration. Similarly, in our research process, we have also delved into the highly esteemed tradition of helping others, which has been upheld from ancient times to the present. Some traditional Chinese proverbs that embody helping behavior are widely known, such as “giving timely help in times of need,” “being generous and charitable,” “selflessly helping others,” “helping others and oneself,” and “doing good deeds and accumulating virtue.” Therefore, our findings undoubtedly challenge the traditional notion of unconditionally admiring all acts of helping behavior, prompting us to take a more cautious approach to the application of this traditional virtue in real life.

Sensitivity analyses indicate that whether it is to enhance life satisfaction or alleviate depression, the existence of functional limitations in individuals is a crucial factor. Only those individuals without functional limitations can benefit from helping behavior. This finding further strengthens our conclusion.

In order to profoundly reveal the positive and substantial benefits of helping behavior on individuals' health, we present a typical case from the Jingmen Blue Sky Social Service Centers (JBSC) in Hubei Province, China [77]. In this case, an individual significantly improved their depressive symptoms by actively participating in volunteer services. Established in 2013, JBSC is the first officially registered social work organization in Jingmen, with the aim of “helping oneself by helping others and sharing the blue sky,” adhering to the concept of “serving society and promoting harmony.” In 2023, JBSC was approved by the Chinese Ministry of Civil Affairs and the Civil Affairs Bureau of Duodao District, Jingmen City, Hubei Province, to implement the “Material + Service” project (project no.: JMKRZB (2023-F14)-D18). In a recent social assistance program conducted by JBSC, a 44-year-old woman who had long been troubled by depression and ovarian cancer, after participating in volunteer services organized by the “Material + Service” project, experienced significant relief from her depressive symptoms. In this case, with the guidance and support of JBSC social workers, she transitioned from a “recipient” to a “helper,” gradually engaging in various volunteer activities in the community, such as environmental maintenance, telephone visits, home visits, and psychological comfort. These activities not only made her feel the warmth and need of society but also helped her find her own value and meaning in helping others. Over time, she became more positive and optimistic, her life satisfaction gradually increased, and her depressive symptoms alleviated. This case fully demonstrates that helping behavior can indeed improve life satisfaction, thereby effectively alleviating depression. This example not only highlights the positive impact of helping behavior on individuals' mental health but also further reinforces the practical value and social significance of such behavior.

However, this study also has several limitations. First, although we referred to previous research to define the indicators for evaluating helping behavior from the CHARLS dataset, CHARLS is a comprehensive database focusing on the health and elderly care of middle-aged and elderly people in China, rather than a specific survey designed to assess helping behavior among the elderly. Therefore, the above indicators may not fully capture the essence of helping behavior. Second, the questionnaires used in the study mainly relied on participations' memory to obtain information, which may lead to the existence of recall bias. Third, despite controlling for established factors influencing depression based on existing literature, residual confounding from unmeasured variables cannot be completely ruled out.

Overall, the results of this study not only deepen our understanding of the mechanisms and treatment methods of depression but also provide valuable guidance for clinical practice, individuals' behavior guidance, and public health policies. From a clinical perspective, we suggest that healthcare professionals incorporate helping behavior into treatment plans and encourage patients to actively engage in helping others. Through this approach, patients can find meaning and value in life, enhance life satisfaction, and indirectly alleviate depression. Additionally, healthcare professionals should monitor life satisfaction as an important indicator during the treatment process to make timely adjustments to treatment plans, ensuring their effectiveness and individualization. From an individual's perspective, on the one hand, we encourage individuals to enhance their sense of happiness and satisfaction by cultivating a positive and optimistic attitude towards life, building good interpersonal relationships, maintaining healthy lifestyle habits, and setting life goals. On the other hand, we encourage individuals to pay attention to their functional status and choose suitable helping behavior accordingly. For individuals with good functional status, participating in helping behavior is a good way to enhance life satisfaction and mental health. For those with limited functionality, they need to find other suitable ways to engage in helping behavior to avoid negative effects. At the public policy level, we recommend that the government take measures to encourage and support the development of helping behavior activities, providing individuals with more opportunities to engage in social interactions and helping behavior. Additionally, the government should expedite the creation of accessible age-friendly environments to ensure that individuals, especially those with functional limitations, can participate and integrate into social life equally, fully, and conveniently. This not only helps improve the public's quality of life and mental well-being but also promotes social harmony and friendliness.

## 5. Conclusions

In the Chinese population, helping behavior can alleviate depression by increasing life satisfaction. However, not everyone benefits from such helping behavior, as there is a “moderation is the key” effect. Particularly, when individuals have functional limitations, helping behavior may have adverse effects on them. These findings not only contribute to a better understanding of the impact of helping behavior on mental health but also provide important references for the development of more personalized psychological health intervention measures.

## Figures and Tables

**Table 1:** Results of linear mixed model for the mediation analysis and moderated mediation analysis (*N* = 7,436).

Variables	Model 1 (depression)		Model 2 (life satisfaction)		Model 3 (depression)		Model 4 (life satisfaction)	
	*B* (95% CI)	*p*	*B* (95% CI)	*p*	*B* (95% CI)	*p*	*B* (95% CI)	*p*
Fixed effects								
Intercept	**7.702** **(6.953, 8.452)**	**<0.001*⁣*^*∗∗∗*^**	**2.817** **(2.722, 2.912)**	**<0.001*⁣*^*∗∗∗*^**	**12.178** **(11.443, 12.913)**	**<0.001*⁣*^*∗∗∗*^**	**2.782** **(2.687, 2.876)**	**<0.001*⁣*^*∗∗∗*^**
Helping behavior	**−0.170** **(−0.313, −0.027)**	**0.020*⁣*^*∗*^**	**0.034** **(0.014, 0.055)**	**<0.001*⁣*^*∗∗∗*^**	−0.116 (−0.256, 0.024)	0.104	**0.048** **(0.026, 0.069)**	**<0.001*⁣*^*∗∗∗*^**
Life satisfaction	—	—	—	—	**−1.614** **(−1.685, −1.543)**	**<0.001*⁣*^*∗∗∗*^**	—	—
IADL	—	—	—	—	—	—	**−0.048** **(−0.058, −0.038)**	**<0.001*⁣*^*∗∗∗*^**
Helping behavior *⁣*^*∗*^ IADL	—	—	—	—	—	—	**−0.047** **(−0.072, −0.023)**	**<0.001*⁣*^*∗∗∗*^**
Random effects								
	Variance (SD)	Correlation	Variance (SD)	Correlation	Variance (SD)	Correlation	Variance (SD)	Correlation
ID (intercept)	9.048 (3.008)	—	0.103 (0.321)	—	7.492 (2.737)	—	0.103 (0.321)	—
Wave (slope)	0.081 (0.285)	−0.11	0.001 (0.031)	0.06	0.068 (0.262)	−0.11	0.001 (0.031)	0.04
Model fits								
Marginal *R*^2^	0.205		0.082		0.259		0.087	
Conditional *R*^2^	0.502		0.327		0.509		0.331	

*Note: B*, unstandardized coefficient; CI, confidence interval; SD, standard deviation; IADL, instrumental activity of daily living. Marginal *R*^2^: fixed effects; Conditional *R*^2^: fixed + random effects. Adjusted for urban–rural disparities, gender, education, age, marital status, chronic disease, fall, pain, self-rated health, indoor air pollution, outdoor air pollution, drink, smoke, sleep duration, internet use.*⁣*^*∗*^*p*  <  0.05,  ^*∗∗∗*^*p* < 0.001. Bold texts are used to highlight values that have statistical significance.

**Table 2:** The mediation effects and conditional indirect effects using the Quasi-Bayesian Monte Carlo method.

Effects	Effect size	LLCI	ULCI
Mediation effects			
Indirect	**−0.055**	**−0.088**	**−0.022**
Direct	−0.118	−0.256	0.024
Total	**−0.173**	**−0.314**	**−0.027**
Conditional indirect effects			
Low IADL (mean − SD)	**−0.114**	**−0.159**	**−0.070**
Average IADL (mean)	**−0.049**	**−0.080**	**−0.020**
High IADL (mean + SD)	0.019	−0.030	0.070

*Note*: IADL, instrumental activity of daily living; LLCI, lower limit of 95% confidence interval; ULCI, upper limit of 95% confidence interval. Bold texts are used to highlight values that have statistical significance.

## Data Availability

The data that support the findings of this study are openly available in CHARLS at https://charls.pku.edu.cn.

## Supplementary Materials

Supplementary Materials The supplementary materials include the results of multicollinearity testing, descriptive statistics, and sensitivity analysis.
