# Supplementary material for: Does Helping Others Always Benefit Health? Longitudinal Evidence on the Relationship between Helping Behavior and Depression: The Mediating Role of Life Satisfaction and the Moderating Effect of IADL
Source: Depress Anxiety. 2024 Jul 25;2024:2304723. doi: 10.1155/2024/2304723 (PMC11918810; doi:10.1155/2024/2304723)
Supplement: Supplementary Materials — The supplementary materials include the results of multicollinearity testing, descriptive statistics, and sensitivity analysis. [file 2304723.f1.docx]

**Table S1.** Results of multicollinearity test

| **Variables** | **GVIF** | **Df** | **GVIF^(1/(2*Df))** |
| --- | --- | --- | --- |
| **Helping behavior** | 1.02 | 1 | 1.01 |
| **Life satisfaction** | 1.07 | 1 | 1.04 |
| **IADL** | 1.10 | 1 | 1.05 |
| **Drink** | 1.16 | 1 | 1.08 |
| **Smoke** | 1.94 | 1 | 1.39 |
| **Sleep** | 1.05 | 2 | 1.01 |
| **Internet use** | 1.21 | 1 | 1.10 |
| **Education** | 1.11 | 2 | 1.03 |
| **Indoor air pollution** | 1.14 | 1 | 1.07 |
| **Outdoor air pollution** | 1.56 | 1 | 1.25 |
| **Chronic disease** | 1.10 | 1 | 1.05 |
| **Fall** | 1.03 | 1 | 1.02 |
| **Pain** | 1.20 | 1 | 1.09 |
| **Self-rated health** | 1.17 | 1 | 1.08 |
| **Marital status** | 1.08 | 1 | 1.04 |
| **Age** | 1.59 | 1 | 1.26 |
| **Urban-rural disparities** | 1.10 | 1 | 1.05 |
| **Gender** | 2.10 | 1 | 1.45 |

Note: GVIF: generalized variance-inflation factors; IADL, Instrumental Activity of Daily Living

**Table S2.** Baseline descriptive statistics of variables

| **Variable** | **Total (n = 7,436)** |
| --- | --- |
| **Depression, mean (SD)** | 8.12 (6.16) |
| **Helping behavior, N (%)** |  |
| No | 6,743 (90.7) |
| Yes | 693 (9.3) |
| **Life satisfaction, mean (SD)** | 3.05 (0.69) |
| **IADL, mean (SD)** | 0.29 (0.79) |
| **Age, mean (SD)** | 56.40 (8.15) |
| **Urban-rural disparities, N (%)** |  |
| Rural | 4,924 (66.2) |
| Urban | 2,512 (33.8) |
| **Gender, N (%)** |  |
| Female | 3,995 (53.7) |
| Male | 3,441 (46.3) |
| **Education, N (%)** |  |
| Primary education | 6,589 (88.6) |
| Secondary education | 760 (10.2) |
| Higher education | 87 (1.2) |
| **Marital status, N (%)** |  |
| Unmarried | 622 (8.4) |
| Married | 6,814 (91.6) |
| **Chronic disease, mean (SD)** | 1.30 (1.32) |
| **Fall, N (%)** |  |
| No | 6,337 (85.2) |
| Yes | 1,099 (14.8) |
| **Pain, N (%)** |  |
| No | 4,965 (66.8) |
| Yes | 2,471 (33.2) |
| **Self-rated health, mean (SD)** | 2.97 (0.89) |
| **Indoor air pollution, N (%)** |  |
| Clean fuel | 2,120 (28.5) |
| Solid fuel | 5,316 (71.5) |
| **Outdoor air pollution, mean (SD)** | 52.17 (17.24) |
| **Drink, N (%)** |  |
| No | 4,935 (66.4) |
| Yes | 2,501 (33.6) |
| **Smoke, N (%)** |  |
| No | 4,613 (62.0) |
| Yes | 2,823 (38.0) |
| **Sleep duration, N (%)** |  |
| < 6 hours / day | 2,103 (28.3) |
| 6-8 hours / day | 3,100 (41.7) |
| ≥ 8 hours / day | 2,233 (30.0) |
| **Internet use, N (%)** |  |
| No | 7,266 (97.7) |
| Yes | 170 (2.3) |

Note: SD, standard deviation; IADL, Instrumental Activity of Daily Living

**Figure S1.** The final moderated mediation model from sensitivity analysis using multiple imputed data (N = 10,828). Note: Unstandardized coefficients are shown; IADL, Instrumental Activity of Daily Living; * *p* < .05, ** *p* < .01, *** *p* < .001

**Figure S2.** The Johnson−Neyman plot of the moderator effect of IADL on the association between helping behavior and life satisfaction from sensitivity analysis using multiple imputed data (N = 10,828). Note: IADL, Instrumental Activity of Daily

**Figure S3.** The Johnson−Neyman plot of the moderator effect of IADL on the association between helping behavior and depression from sensitivity analysis using multiple imputed data (N = 10,828). Note: IADL, Instrumental Activity of Daily

**Figure S4.** The final moderated mediation model from sensitivity analysis among participants aged 50 and above (N = 5,535). Note: Unstandardized coefficients are shown; IADL, Instrumental Activity of Daily Living; * *p* < .05, ** *p* < .01, *** *p* < .001

**Figure S5.** The Johnson−Neyman plot of the moderator effect of IADL on the association between helping behavior and life satisfaction from sensitivity analysis among participants aged 50 and above (N = 5,535). Note: IADL, Instrumental Activity of Daily
